# Spondylarthrite ankylosante au Sénégal: aspects épidémiologiques, diagnostiques, thérapeutiques et évolutifs au Centre Hospitalier Universitaire Aristide LeDantec de Dakar

**DOI:** 10.11604/pamj.2021.40.15.29525

**Published:** 2021-09-06

**Authors:** Abbasse Abba, Moustapha Niasse, Ladji Mohamed Diaby, Hassan Ali

**Affiliations:** 1 Service de Rhumatologie, Centre Hospitalier Universitaire Aristide LeDantec, Dakar, Sénégal

**Keywords:** Spondylarthrite ankylosante, prédominance féminine, clinique, HLA B27, biothérapie, évolution, Sénégal, Ankylosing spondylitis, female predominance, clinical, HLA B27, biotherapy, evolution, Senegal

## Abstract

**Introduction:**

la spondylarthrite ankylosante (SA) est une maladie évolutive, pouvant s'aggraver par une invalidité. Le but de notre étude est de décrire les aspects épidémiologiques, diagnostiques, thérapeutiques et évolutifs de la SA dans le service de rhumatologie du Centre Hospitalier Universitaire Aristide LeDantec à Dakar.

**Méthodes:**

il s´agissait d´une étude transversale descriptive et analytique, avec un recueil de données à la fois prospectif et rétrospectif sur une période de 8 ans, entre janvier 2012 et décembre 2020, sur des patients atteints de SA de diagnostic établi en accord avec les critères diagnostiques d´Amor, de l´ESSG (European Seronegative Spondylarthropathy Group), d´ASAS (Assessment of Spondyloarthritis International Society) et les critères de New York modifié pour la SA. Les données ont été recueillies par le biais d'un questionnaire structuré et analysées à l´aide du logiciel SPSS25 (Statistical Package for the Social Sciences).

**Résultats:**

six cent quarante-sept (647) patients ont répondu aux critères d´inclusions; 414 femmes et 233 hommes soit un sex ratio de 1,77F/1H. Différentes formes symptomatiques étaient obtenues: les formes axiales (55,65%), les formes mixtes (44,35%) et les formes systémiques avec manifestation extra-articulaires: uvéites (12,21%), insuffisance aortique (5,71%) et maladie fibrobulleuse des poumons (3,86%). Soixante pourcent (60%) des patients étaient sous antiinflammatoires non stéroïdiens (AINS), 47% sous méthotrexate et 0,92% sous biothérapie. Les indices d´activité de la maladie, les indices fonctionnels et les indices de la qualité de vie ont permis le suivi de la maladie.

**Conclusion:**

nos résultats ont montré une prédominance féminine. Les formes axiales étaient les plus représentées. Plus de la moitié de nos patients étaient sous antiinflammatoires, 47% sous méthotrexate et 0,92% sous biothérapie. Cette étude a permis de comprendre le poids de la SA dans les spondyloarthrites et son évolution dans le temps.

## Abstract (en)

**Introduction:**

ankylosing spondylitis (AS) is a progressive disease, which can result in disability. The purpose of this study is to describe the epidemiological, diagnostic, therapeutic and evolutionary features of AS in the Department of Rheumatology of the Hospital Center University Aristide Le Dantec, Dakar.

**Methods:**

we conducted a descriptive and analytical cross-sectional study. Data were collected on a prospective and retrospective basis over a period of 8 years, between January 2012 and December 2020. Patients were diagnosed with AS on the basis of ESSG (European Seronegative Spondylarthropathy Group) and Amor diagnostic criteria, ASAS (Assessment of Spondyloarthritis International Society) criteria and modified New York criteria. Data were collected by a structured questionnaire and analyzed using the SPSS25 (Statistical Package for the Social Sciences) software.

**Results:**

six hundred forty-seven patients met the inclusion criteria (414 women and 233 men) with a sex ratio of 1.77F/1M. Different symptomatic cases were found: axial disease (55.65%), mixed disease (44.35%) and systemic disease with extra-articular manifestations including uveitis (12.21%), aortic insufficiency (5.71%) and fibrobullous lung disease (3.86%). Sixty percent of patients were receiving non-steroidal anti-inflammatory drugs (NSAIDs), 47% methotrexate, and 0.92% biotherapy. Disease activity index, functional index and quality of life index enabled disease monitoring.

**Conclusion:**

our results show that there was predominance in women. Patients were mostly affected by axial spondyloarthritis. More than half of our patients were treated with anti-inflammatory, 47% with methotrexate and 0.92% with biotherapy. This study highlights that the features of ankylosing spondylitis (AS) are a burden to the patient with spondyloarthritis and disease progression over time.

## Introduction

La spondylarthrite ankylosante (SA) est le chef de file d´un groupe de rhumatismes inflammatoires communément appelés spondyloarthrites incluant les arthrites réactionnelles, l´arthrite psoriasique, les spondylarthrites indifférenciées, les rhumatismes associés aux entérocolopathies (Rectocolite hémorragique RCH, maladie de Crohn…), ainsi que certaines spondylarthrites juvéniles. Ces pathologies sont caractérisées par des manifestations cliniques et radiologiques communes telles que des lombalgies inflammatoires, une atteinte des enthèses et des articulations périphériques, ainsi que des manifestations extra-articulaires touchant la peau, le cœur, les yeux et les poumons. Elles présentent également le même terrain génétique prédisposant, dominé par l´antigène HLA-B27 (*Human Leukocyte Antigen* B27) [1-4]. Au cours de l´évolution de la maladie, des dommages structuraux et fonctionnels peuvent survenir, telle que la rigidification de la colonne, avec pour conséquence une altération de la qualité de vie. La spondylarthrite ankylosante touche le sujet jeune et prédomine chez le sexe masculin.

**Contexte rationnel:** comme dans toutes les grandes villes de l´Afrique subsaharienne, la plupart des spécialistes est concentrée dans les centres urbains et Dakar ne fait pas exception, à ce titre le diagnostic et la prise en charge de ces affections est rendu difficile. Une meilleure connaissance des aspects épidémiologiques, diagnostiques, thérapeutiques et évolutifs de la SA permettra de prévenir et d'améliorer la prise en charge de la maladie.

**Objectifs:** le but de ce travail était de décrire les aspects épidémiologiques, diagnostiques, thérapeutiques et évolutifs de la SA au CHU Aristide LeDantec à Dakar au Sénégal.

## Méthodes

**Cadre de l'étude:** il s´agit d´une étude transversale descriptive et analytique, avec un recueil de données à la fois prospectif et rétrospectif. Nous avons colligé 647 cas de spondylarthrite ankylosante dans le service de Rhumatologie du CHU Aristide LeDantec à Dakar, durant la période d´étude, entre le 1^er^ janvier 2012 et le 31 décembre 2020.

**Contexte organisationnel:** cette étude a concerné tous les patients dont les dossiers médicaux étaient complets. Des fiches d´exploitations préétablies ont été remplies regroupant les paramètres épidémiologiques, cliniques, thérapeutiques et évolutifs.

**Participants:** les patients inclus sont hospitalisés ou suivis en ambulatoire dans le service de Rhumatologie du CHU Aristide LeDantec à Dakar, pour spondylarthrite ankylosante de diagnostic établi selon les critères d´Amor, de l´ESSG (*European Seronegative Spondylarthropathy Group*), d´ASAS (*Assessment of Spondyloarthritis International Society*) et les critères de New York modifié pour la SA.

### Variables étudiées

1) Les données sociodémographiques (l´âge, le sexe, la situation matrimoniale, la scolarité, la profession, l´origine géographique, l´ethnie, les antécédents familiaux de SpA, le mode de vie (alcool, tabac)). 2) Les données cliniques (mode de début, durée d´évolution de la maladie, atteinte des hanches, manifestations extra-articulaires). 3) Les données biologiques: le syndrome inflammatoire biologique est défini par l'élévation d'au moins 2 protéines de l'inflammation (protéine C-réactive (CRP), albumine, α1-globulines, α2-globulines, β-globulines, γ-globulines, fibrinogène, protéine totale) ou de la vitesse de sédimentation (VS), et d´une protéine de l'inflammation; la recherche de l´antigène HLAB27. 4) Les données radiologiques (Classification de FORESTIER, Score de mSASS: *modified Stoke Ankylosing Spondylitis Spinal Score*, index de GENANT). 5) Les données thérapeutiques: les antalgiques selon les paliers de l´OMS, les coantalgiques, les corticoïdes, les AINS, les DMARD synthétique (salazopyrine, méthotrexate, léflunomide…), les innovants notamment les anti-TNFa. Ainsi que le recours à des infiltrations cortisoniques, à la kinésithérapie et à la chirurgie orthopédique. 5) Les données sur l´indice de l´activité de la maladie (BASDAI : *Bath Ankylosing Spondylitis Disease Activity Index*, ASDAS: *Assessment in Ankylosing Spondylitis-Endorsed Disease Activity Score*). 6) Les données sur l´indice de retentissement fonctionnel (BASFI: *Bath Ankylosing Spondylitis Functional Index*). 7) Les données sur l´indice de la qualité de vie: questionnaires *Short Form* 36 (SF-36), *Nottingham Health Profile* (NHP) et ASQoL: *Ankylosing Spondylitis Quality of life*.

**Sources de données:** c’est une étude documentaire du dossier médical de chaque patient.

**Biais:** comme il s'agissait d'une étude en bonne partie rétrospective, il y avait des données manquantes. Ces patients étaient exclus des analyses pour les variables dont les données manquaient.

**Taille de l´étude:** le nombre de cas admis au cours de la période d'étude a déterminé la taille de l'échantillon.

**Les variables quantitatives et qualitatives:** les données qualitatives ont été décrites en nombre et en pourcentage. Les données quantitatives ont été décrites par des moyennes et des écart-types.

**Méthodes statistiques:** les données ont été recueillies à l´aide de questionnaires sur la base des dossiers médicaux, puis des examens de suivi-contrôle. Elles ont été saisies et analysées à l´aide des logiciels Microsoft Office Word 2007, Access 2007 et SPESS version 25. Le test de khi-deux de Pearson a été utilisé pour la comparaison des variables qualitatives et le test de Yates s'il y'a lieu. Pour les effectifs inférieurs à 5, nous avons utilisé le test de Fisher. Les différences constatées ont été considérées comme significatives pour un p < 0,05 avec un intervalle de confiance à 95%.

**Considérations éthiques:** le fait de remplir le questionnaire a été considéré comme une procuration pour le consentement éclairé. Le principe de confidentialité était rigoureusement observé lors de la collecte, de la saisie et de l'analyse des données en utilisant l´anonymat.

## Résultats

### Caractéristiques sociodémographiques des participants

Nous avons colligé 9262 observations de patients dans le service de rhumatologie, durant la période d'étude allant du 1^er^ janvier 2012 au 31 décembre 2020, dont 647 avaient une spondylarthrite ankylosante soit une prévalence hospitalière de 0,87% et une incidence annuelle de 1,08 pour 100.000 personne-année. La tranche d´âge la plus représentée était la tranche de [41 - 50] ans avec un pourcentage de 26,7%; La prédominance féminine était notée avec 414 femmes (64%) et 233 hommes (36%) soit un sex ratio de 1,77F/1H. L´âge moyen de nos patients était de 47,28 ± 15,49 au moment du diagnostic avec des extrêmes de 14 et 92 ans. L´âge de début était avant 50 ans chez 49,93% et un début tardif chez 36,25%. La forme juvénile (16 ans et moins) était présente avec 9 cas soit 1,39% et la forme gériatrique (65 ans et plus) était représentée avec 99 patients soit 15,30%. Dans notre série d'étude, 49,3% seraient des personnes sans activités professionnelles, 13,22% avaient une profession intermédiaire et 12,34% étaient des artisans, commerçants et chefs d'entreprise. Le délai moyen du diagnostic était de 5,65 ans avec des extrêmes allant de 1 mois à 40 ans.

### Caractéristiques cliniques de la population

Le motif de consultation le plus fréquent dans notre série était dominé par les douleurs pelvi-rachidiennes (66,2%) et les atteintes articulaires périphériques (35,1%) prédominant aux membres inférieurs. L´installation de la douleur était progressive chez 65% de nos patients. La douleur était inflammatoire dans 45,75% des cas et dans 87,56% des cas, la douleur était chronique. Le dérouillage matinal était présent chez 36,25% des patients avec une durée environ une heure. L´atteinte axiale était dominée par l´atteinte du rachis lombaire avec 98,76%, 49,76% avait une cervicalgie et 41,73% avait une atteinte pelvienne. 33,23% de nos patients avaient une oligoarthrite asymétrique et 13,13% une polyarthrite asymétrique prédominant aux membres inférieurs. Les synovites était présent chez 299 patients soit 46,21%. Les articulations les plus concernées étaient les genoux avec 44,35%, les chevilles avec 36,63% et les pieds avec 25,81%. Nous avons noté la présence de coxite clinique dans 18,31% des cas. L´atteinte enthésique était présente avec 27,82% de fessalgie, 25,65% de talalgie antérieure et 21,02% de talalgie postérieure. Grâce aux critères d´Amor, de l´ESSG, d´ASAS et les critères de New York modifié pour la SA, différentes formes symptomatiques étaient obtenues: les formes axiales (360 cas soit 55,65%) suivies des formes mixtes (287 cas soit 44,35%) et les formes systémiques avec manifestation extra-articulaires dont : uvéites (79 cas soit 12,21%), insuffisance aortique (37 cas soit 5,71%) et maladie fibrobulleuse des poumons (25cas soit 3,86%). Parmi les pathologies associées les plus fréquentes étaient le Syndrome de Gougerot Sjögren (5,8%) et la Polyarthrite Rhumatoïde (1,4%) dans les maladies auto-immunes ; Concernant les maladies auto-inflammatoires associées les plus fréquentes figuraient la goute (1,7%) et la chondrocalcinose (0,3%) ; Les maladies dégénératives associées les plus fréquentes étaient l'arthrose (9,9%) et l'ostéoporose (6,8%) ; Les comorbidités les plus fréquemment associées aux SA dans notre série étaient l´hypertension artérielle (10,1%) et le diabète (4,3%).

### Caractéristiques paracliniques de la population

Un syndrome inflammatoire biologique était présent chez 534 patients au moment du diagnostic. La Vitesse de Sédimentation (VS) était élevée chez 398 patients avec une moyenne de 40,78 mm (extrêmes 0 et 190). La CRP était élevée chez 325 patients avec une moyenne de 26,48 mg/l (extrêmes 6 et 192). L´Ag HLA-B27 recherché chez 401 patients était positif chez 208 (51,87% des cas). Le sous-typage HLA-B27 n'était pas réalisé dans notre série. Sur le plan radiologique: une sacro-iléiite était retrouvée soit sur une radiographie ou soit sur un scanner du bassin dans 589 cas soit 91,03%, évaluée par la classification de Forrestier avec une atteinte supérieure au stade 2 de FORESTIER dans 75,86% des cas. Le mSASSS moyen était de 24,57 avec des extrêmes de 0 à 72. Le score mSASSS a permis d'évaluer le rachis lombaire chez 237 patients et le rachis cervical chez 72 patients. L'index de GENANT prenant en compte le rachis dorsal et lombaire a permis le suivi structurel de l'ostéoporose chez 158 patients dont 5 avait une fracture vertébrale et 12 une déformation vertébrale modérée.

### Caractéristiques de prise en charge thérapeutique de la population

Les antalgiques (Paracétamol et/ou Tramadol) ont été utilisés chez 91,21%, 60% recevaient des antiinflammatoires non stéroïdiens. 47% étaient sous Méthotrexate, alors que 13% étaient sous Sulfasalazine. Dans notre série, 0,92% étaient sous biothérapie (anti-TNFalpha). Chez 3,4% des cas, nous avons eu recours à la kinésithérapie, alors que 0,30% de nos patients ont été adressés en traumatologie pour prothèse totale de hanche et 0,30% autre pour une ostéotomie rachidienne.

### Caractéristiques évolutives de la population

L'indice d´activité a été évalué par BASDAI et ASDAS-CRP, la moyenne de BASDAI était de 5 avec des extrêmes de [0 - 9,55] et de 2,53 avec des extrêmes de [0,10 - 9] pour l'ASDAS-CRP au début du traitement. Cette moyenne a considérablement diminué tout au long du traitement ainsi donc on notait pour le BASDAI une moyenne de 2,61 avec des extrêmes de [0,40 - 7] au 3^e^ mois et de 2,76 avec des extrêmes de [0,20 - 7,7] au 6^e^ mois et pour l´ASDAS-CRP elle était de 1,61 avec des extrêmes de [1 - 3,6] au 3^e^ mois et de 1,78 avec des extrêmes de [0,94 - 6] au 6^e^ mois. L'indice de retentissement fonctionnel a été évalué par BASFI, la moyenne de BASFI était de 5,03 avec des extrêmes de [0 - 9] au début du traitement. Cette moyenne a considérablement diminué tout au long du traitement ainsi donc on notait une moyenne de 2,35 avec des extrêmes de [0 - 5,2] au 3^e^ mois et de 2,58 avec des extrêmes de [0 - 7] au 6^e^ mois. L'indice de la qualité de vie a été évalué par NHP, SF36 et ASQoL. On notait un score moyen pour l'ASQOL sur 18 de 12,97 avec des extrêmes allant de [3 - 17] au début du traitement. Le score moyen de l'ASQoL était au 3^e^ mois du traitement à 4,39 avec des extrêmes allant de [2 - 12] et au 6^e^ mois du traitement à 3,88 avec des extrêmes allant de [2 - 12]. Dans la SF36 au début du traitement: l'activité physique (67,20%), les douleurs physiques (65,52%), la santé perçue (60,60%) et le fonctionnement social (59,73%) étaient les plus représentés. S'agissant de la NHP au début du traitement : la douleur (66,70%), l'énergie (55,52%) et la mobilité (31,89%) étaient les plus représentés. Les scores de SF36 (Tableau 1), de NHP (Tableau 2) et d'ASQOL (Tableau 3) étaient importants à l'initiation du traitement et relativement diminués au troisième et au sixième mois du traitement.

**Tableau 1:** répartition des patients selon le SF36

SF36	M3	M6
**Activité physique**	35,94	41,05
**Limitations dues à l´état physique**	23,40	23,05
**Douleurs physiques**	38,60	41,77
**Santé perçue**	37,43	39,66
**Vitalité**	26,95	29,23
**Fonctionnement social**	30,39	34,19
**Santé psychique**	29,37	35,28
**Limitations dues à l´état psychique**	23,69	21,59
**PCS**	35,95	35,63
**MCS**	28,24	28,32

**Tableau 2:** résumé des valeurs du NHP

NHP	M3	M6
Mobilité	16,12	14,57
Douleur	17,77	16,03
Isolation sociale	6,24	1,85
Réaction émotionnelle	3,43	2,43
Energie	34,42	30,18
Sommeil	22,95	20,56

**Tableau 3:** résumé des valeurs de l´ASQoL

ASQoL	M3	M6
**Limitation des déplacements**	38,2%	37,4%
**Envie de pleurer à cause de la maladie**	24,6%	22,2%
**Difficulté à s´habiller**	25,1%	24,4%
**Fatigue au cours des activités de la vie quotidiennes**	24,5%	23,7%
**Problèmes de sommeils**	17,6%	15,8%
**Vie sociale**	18,4%	17,5%
**Fatigue continue**	32,6%	30,9%
**Se reposer au cours du travail**	33,7%	32,3%
**Douleur insupportable**	27,7%	36,9%
**Raideur matinale**	26,4%	25,2%
**Limitation des activités de la vie quotidienne**	23,8%	22,8%
**Se fatiguer rapidement**	32,9%	30,6%
**Frustration**	24,6%	23,1%
**Douleur continue**	30,3%	28,2%

## Discussion

### Aspects épidémiologiques

**Figure 1:**
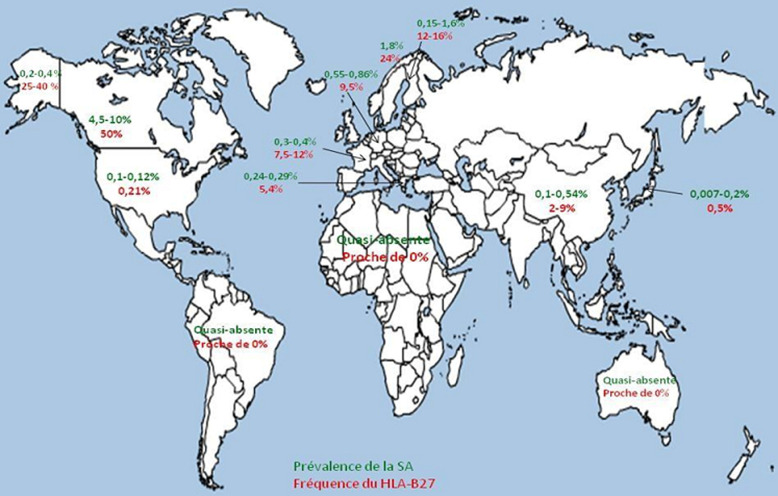
gradient décroissant Nord/Sud de la prévalence de la SA et de la fréquence du HLA-B27

De nombreuses études ont évalué les taux d´incidence et de prévalence de la SA dans les diverses régions du globe, mais les différences méthodologiques rendent difficile la comparaison et l´interprétation des résultats entre les pays ou pour un même pays. Trois principaux facteurs influencent ces taux: La sélection de la population à étudier. Les paramètres de dépistage des patients atteints de la maladie. La variation fréquente au sein d´une même population, pour certains groupes ethniques, de la prévalence de la SA [1, 5]. Ce dernier facteur serait dû au fait que la prévalence et l´incidence de la SA seraient en étroite corrélation avec la fréquence de l´antigène HLA-B27 dans les différentes populations. Ainsi, les peuples chez lesquels un taux élevé d´antigène HLA-B27 est retrouvé, ont une incidence ou une prévalence plus élevée pour la maladie [5-8]. Les différences géographiques et ethniques dans la répartition de l´antigène HLA-B27 suggèrent l´existence d´un gradient décroissant Nord-Sud pour ce qui est de la prévalence et de l´incidence de la maladie [9, 10] (Tableau 4 et Figure 1). Les prévalences les plus élevées de l´HLA-B27 et de la SA sont retrouvées dans les populations natives du cercle polaire arctique et des régions subarctiques de l´Eurasie et d´Amérique du Nord [8]. La SA est quasi-absente en Amérique du Sud, en Australie et en Afrique, même si en Afrique occidentale, la fréquence du HLA-B27 est non négligeable (2 à 9%). La maladie serait même absente chez les sujets porteurs de l´allèle B*2705 dont l´association avec la SA a été clairement prouvée. Ceci prouverait qu´outre le HLA-B27, d´autres facteurs sont nécessaires au développement de la maladie. Des résultats similaires ont aussi été trouvés dans la population afro-américaine. Ainsi la proportion de personnes porteuses du HLA-B27 dans cette population serait de l´ordre de 2 à 4%, contre 8% dans la population caucasienne. De plus, seulement 50% des sujets spondylarthritiques afro-américains seraient HLA-B27 positifs contre 90% dans la population caucasienne [8]. Dans notre étude la tranche d´âge la plus représentée était la tranche de [41 - 50] ans avec un pourcentage de 26,7%. L´âge moyen de nos patients était de 47,28 ± 15,49 au moment du diagnostic avec des extrêmes de 14 et 92 ans. Ce résultat est supérieur à ceux de Awada *et al*. [11] et Jabbouri *et al*. [12] qui ont retrouvé respectivement un âge moyen de 37,3 ans et 32 ans. La prédominance féminine était notée dans notre travail avec 414 femmes (64%) et 233 hommes (36%) soit un sex ratio de 1,77F/1H. Ce résultat diffère à ceux de Awada [11] et Jabbouri [12] pour qui le sexe masculin est le plus représenté.

**Tableau 4:** récapitulatif des prévalences et incidences de la SA dans le monde, ainsi que de la fréquence du HLA-B27 [6, 7, 9]

Population	Prévalence de la S.A (%)	Incidence de la S.A (/100 000)	Fréquence du HLA B27 (%)
**Norvège Laponie**	0,41; 1,1 -1,4 1,8	7,26 - 10	14 - 16 24
**Finlande**	0,15 - 1,6	6,9	12 - 16
**Grèce**	0,24 - 0,29	1,5	5,4
**Turquie**	0,14 - 0,49		7 - 14
**France**	0,3 - 0,4		7,5 - 12
**Allemagne**	0,55 - 0,86		9,5
**USA**	0,10 - 0, 12		0,21
**Japon**	0,007 - 0,2	0,48	0,5
**Chine**	0,1 - 0,4 Ou 0,19 - 0,54		2 - 9
**Esquimaux d´Alaska**	0,2 - 0,4		25 - 40
**Indiens Haïda (Ouest canadien)**	4,5 - 10		50
**Australie Amérique du sud**	Quasi Absente		Proche de 0
**Afrique sub-saharienne Ouest Africain**	Quasi Absente		Inférieure à 1 2 - 9

### Aspects diagnostiques

Le motif de consultation le plus fréquent dans notre série était dominé par les douleurs pelvi-rachidiennes (66,2%) et les atteintes articulaires périphériques (35,1%) prédominant aux membres inférieurs, ce qui conforme à la littérature dominée par la polyarthrite asymétrique chronique (23,1%). L´installation de la douleur était progressive chez 65% de nos patients. La douleur était inflammatoire dans 45,75% des cas et dans 87,56% des cas, la douleur était chronique. Awada [11] a retrouvé ce même caractère chronique et inflammatoire de la douleur. Cliniquement dans notre étude l´atteinte articulaire périphérique était présente avec 33,23% d'oligoarthrite asymétrique et 13,13% de polyarthrite asymétrique prédominant aux membres inférieurs associée à une synovite dans 46,21%. Les articulations les plus concernées étaient les genoux avec 44,35%, les chevilles avec 36,63% et les pieds avec 25,81%. Nous avons noté également la présence de coxite clinique dans 18,31% des cas. Ce résultat est inférieur à ceux d´Awada [11] et Jabbouri [12] qui ont trouvé une coxite clinique dans, respectivement, 27,55% et 31,3%, par contre nos résultats sont supérieurs à ceux de Gerard *et al*. [13] qui ont obtenu 12% de coxite clinique. Dans notre étude l´atteinte axiale était dominée par l´atteinte du rachis lombaire avec 98,76%, 49,76% avait une cervicalgie et 41,73% avait une atteinte des sacro-iliaques.

Nos résultats sont supérieurs à ceux d´Awada [11] et Jabbouri [12] qui ont trouvé une atteinte lombaire dans respectivement, 82,65% et 83,2%. Awada [11] et Jabbouri [12] trouvèrent une atteinte des sacro-iliaques dans, respectivement 56,52% et 63,8%, ce qui est supérieur à notre étude. Dans notre série la plupart des patients (92,31%) présentait des signes extra-articulaires. Les uvéites (79 cas soit 12,21%) représentaient l´atteinte extra-articulaire la plus fréquente. Ce résultat est similaire à celui de Jabbouri [12] pour qui l'uvéite est le signe extra-articulaire le plus présent. Alors que Awada [11] retrouve la diarrhée et Gerard [13] le psoriasis. Le dérouillage matinal était présent chez 36,25% de nos patients avec une durée environ une heure. Awada [11] trouve un dérouillage matinal dans environ 66,19% des cas. L´atteinte enthésique dans notre étude était présente avec 27,82% de fessalgie, 25,65% de talalgie antérieure et 21,02% de talalgie postérieure. Awada [11] trouve une enthésite dans 54,08% des cas avec un siège prédominant au talon (42,86%). Gerard [13] par contre, trouve 12% d´enthésite active.

Parmi les pathologies associées, les plus fréquentes dans notre série étaient notées le SGS (5,8%) et la PR (1,4%) dans les maladies auto-immunes associées ; concernant les maladies auto-inflammatoires associées figuraient la goute (1,7%) et la chondrocalcinose (0,3%) étaient les plus fréquentes; les maladies dégénératives associées à la SA, les plus représentées étaient l'arthrose (9,9%) et l'ostéoporose (6,8%) ; les comorbidités les plus fréquemment associées à la SA dans notre série étaient l'HTA (10,1%) et le diabète (4,3%). Di Fazano [14] dans sa série de 41 cas de SA et 102 témoins, notait l´association avec un SGSP chez 31,7% des patientes, tandis que Benamour [15] rapporte cette association chez 1 seul de ses patients. La cohorte DESIR était caractérisée par une faible prévalence des comorbidités [16]. La physiopathologie de ces comorbidités n´est pas claire, mais serait probablement liée au processus inflammatoire de la maladie, à ses traitements et en particulier à la consommation au long cours d´AINS ou d´autres facteurs tels que l´immobilisation du rachis en raison de l´ankylose.

Dans notre travail la vitesse de sédimentation était élevée chez 398 (61,51%) patients avec une moyenne de 40,78 mm (extrêmes 0 et 190). Résultat inférieur à celui de Awada [11] qui trouve une Vitesse de sédimentation élevée chez 100,0% des patients et Gerard [13] qui trouve une vitesse de sédimentation élevée dans 66,0% des cas. La CRP était élevée chez 325 (50,23%) de nos patients avec une moyenne de 26,48 mg/l (extrêmes 6 et 192). Ce résultat est inférieur à celui de Gerard [13] qui trouve une CRP positive dans 54,0% des cas. Une sacro-iléiite était retrouvée soit sur une radiographie ou soit sur un scanner du bassin chez 589 de nos patients soit 91,03%, évaluée par la classification de Forestier avec une atteinte supérieure au stade 2 dans 75,86% des cas. Ce résultat est supérieur à ceux de Awada [11] et Jabbouri [12] qui ont trouvé respectivement 78,02% et 93,2% d´atteinte des sacro-iliaques radiologiquement, avec une atteinte supérieure au stade 2 de Forestier dans 70,32% des cas pour Awada [11]. Ceci pourrait s´expliquer par le fait que la radiographie standard ne constitue pas le moyen le plus efficace pour la détection des signes inflammatoires en phase débutante. Le score moyen de mSASSS dans notre série était de 23,32%. C´est un score peu sensible pour les formes débutantes et mieux adapté aux formes avec une longue évolution comme le cas de notre étude. La vitesse de progression des ossifications et la variabilité de la progression ont été analysées avec le score mSASSS. Cette vitesse de progression est lente, de l´ordre de 1 à 1,5 points mSASSS en deux ans pour des formes ayant au moins dix ans d´évolution, mais avec une grande variabilité de progression [17].

### Aspects thérapeutiques

Les AINS dans notre étude étaient prescrits dans 58% des cas, un traitement à base de MTX a été prescrit dans 45,5% et la salazopyrine dans 11,5%. Ce résultat est en accord avec celui de Frikha qui rapportait dans sa série, qu´à côté des AINS, le MTX était prescrit dans 32,5% des cas. En effet, les AINS (en l´absence de contre-indication) représentent le traitement de référence et de première ligne dans la prise en charge des SpA. Le choix de l´AINS sera notamment basé sur l´appréciation des risques cardiovasculaires, gastro-intestinaux et rénaux. L´utilisation fréquente des DMARDs pourrait s´expliquer d´une part, par la présence d´atteinte périphérique dû à la prédominance féminine dans notre série et d´autre part, par l´accès difficile aux biothérapies dans notre contexte (utilisé chez six patients soit 0,78%). Le traitement physique est d´un intérêt important dans la SpA afin de préserver la mobilité, la souplesse rachidienne et l´ampliation thoracique correcte [18].

### Aspects évolutifs

Dans notre série le score moyen de SF36 était important à l'initiation du traitement puis diminue au troisième mois pour se stabiliser au sixième mois, et cela, en particulier pour l´activité physique (67,20), la limitation due à l´état physique (59,18) et la douleur physique (65,52); il en est de même pour le NHP: mobilité (31,89) et douleur (66,70). Ce résultat est différent de celui de la population générale avec un score moyen de SF36 pour l´activité physique de 84,45%, et de 81,21% pour la limitation due à l´état physique. Cela traduit le degré d´altération de la qualité de vie au cours de la SA [16]. Nos données sur l'ASQoL à l'initiation du traitement se rapprochent de celles de l'équipe tunisienne dirigée par Hamdi *et al*. [19] qui ont obtenu une limitation des déplacements à 63,6% vs 69,3% (notre étude), une douleur insupportable à 54,4% vs 57,7% (notre étude), une raideur matinale à 53,5% vs 52,7% (notre étude) et une limitation des activités de la vie quotidiennes à 44,4% vs 46,8% (notre étude). Par contre leur étude étant étalée sur 10 jours, nos données sur l'ASQoL au 3^e^ et au 6^e^ mois de traitement ne sont pas confrontées aux leurs. Le retentissement de la maladie était important, tant sur le plan fonctionnel que de l´activité de la maladie avec une valeur moyenne avant traitement du BASFI à 5,03 et celle du BASDAI à 5. Nos données sont comparables à celle de la Tunisie ou la moyenne de BASFI et BASDAI était respectivement de 4,7 et de 5 [20]. Par contre, elle était légèrement inférieure à celle de Gérard *et al*. [13] avec une moyenne de BASFI à 5,9 et de BASDAI à 5,96. Cependant, elle est supérieure à celle de la cohorte Desir (BASFI 3,4) [16]. La valeur moyenne ASDAS était de 2,53, ce qui était proche de celle de la cohorte Desir [16]. Nous avons retrouvé une corrélation entre BASFI, BASDAI, ASDAS, ce qui va dans le sens des données de la littérature. Apres trois mois de traitement, la valeur moyenne de BASFI, de BASDAI et d'ASDAS étaient respectivement de 2,35, de 2,61 et de 1,61 Apres six mois de traitement, la valeur moyenne de BASFI, de BASDAI et d'ASDAS étaient respectivement de 2,58, de 2,76 et de 1,78.

**Limites de l'étude:** ce travail présente quelques limites à la généralisation de ses déductions dont l'aspect rétrospectif et documentaire de collecte de données susceptible de faire perdre des informations importantes mais ne figurant pas sur les dossiers des patients. Autres limites l´Ag HLA-B27 n'a été recherché que chez 401 patients et la biothérapie n´a été utilisée que chez six patients.

## Conclusion

Les données épidémiologiques, diagnostiques, thérapeutiques et évolutives nous ont permis de comprendre le poids de la spondylarthrite ankylosante et son évolution dans le temps. Les nouveaux critères de classifications vont surement modifier l´épidémiologie de la spondylarthrite ankylosante en Afrique sub-saharienne longtemps sous-estimé, au cours des prochaines études.

### Etat des connaissances sur le sujet

- La spondylarthrite ankylosante entraîne par sa fréquence et son retentissement sur la capacité fonctionnelle un handicap important et une altération de la qualité de vie des patients.

### Contribution de notre étude à la connaissance

- Notre recherche a permis de retrouver une prédominance féminine et un peu moins de la moitié de nos patients sont sans activités professionnelles ;
- Un syndrome inflammatoire biologique et l´antigène HLAB27 présents chez plus de la moitié de nos patients ;
- Des scores de SF36, de NHP et d'ASQOL importants à l'initiation du traitement et relativement diminués au troisième et au sixième mois du traitement.

## Remerciements

L'auteur correspondant tient à remercier l'ensemble du personnel du service de rhumatologie et des DES de rhumatologie pour leurs disponibilités et leurs encouragements.
